# High Sensitivity Monitoring of VOCs in Air through FTIR Spectroscopy Using a Multipass Gas Cell Setup

**DOI:** 10.3390/s22155624

**Published:** 2022-07-27

**Authors:** Annalisa D’Arco, Tiziana Mancini, Maria Chiara Paolozzi, Salvatore Macis, Lorenzo Mosesso, Augusto Marcelli, Massimo Petrarca, Francesco Radica, Giovanna Tranfo, Stefano Lupi, Giancarlo Della Ventura

**Affiliations:** 1 National Institute for Nuclear Physics Laboratori Nazionali Frascati (INFN-LNF), Via E. Fermi 54, 00044 Frascati, Italy; augusto.marcelli@lnf.infn.it; 2 Department of Physics, University of Rome ‘La Sapienza’, P.le A. Moro 2, 00185 Rome, Italy; tiziana.mancini@uniroma1.it (T.M.); salvatore.macis@uniroma1.it (S.M.); stefano.lupi@uniroma1.it (S.L.); 3 National Institute for Nuclear Physics Section Rome1, P.le A. Moro 2, 00185 Rome, Italy; massimo.petrarca@uniroma1.it; 4 Department of Science, University Rome Tre, Largo San Leonardo Murialdo 1, 00146 Rome, Italy; paolozzi.1698739@studenti.uniroma1.it (M.C.P.); lorenzo.mosesso@gmail.com (L.M.); giancarlo.dellaventura@uniroma3.it (G.D.V.); 5 Rome International Centre for Materials Science Superstipes, Via dei Sabelli 119A, 00185 Rome, Italy; 6 Department of Basic and Applied Sciences for Engineering (SBAI), University of Rome ’La Sapienza’, Via Scarpa 16, 00161 Rome, Italy; 7 Department of Engineering and Geology, University Gabriele d’Annunzio Chieti-Pescara, Via dei Vestini, Campus Universitario, 66100 Chieti, Italy; francesco.radica@unich.it; 8 Department of Occupational and Environmental Medicine, Epidemiology and Hygiene, INAIL, Monte Porzio Catone, 00078 Rome, Italy; g.tranfo@inail.it; 9 INGV, Via di Vigna Murata 605, 00143 Rome, Italy

**Keywords:** VOCs, FTIR, sensor, accuracy, ppm_v_, styrene, isopropanol, acetone, ethanol

## Abstract

Human exposure to Volatile Organic Compounds (VOCs) and their presence in indoor and working environments is recognized as a serious health risk, causing impairments of varying severities. Different detecting systems able to monitor VOCs are available in the market; however, they have significant limitations for both sensitivity and chemical discrimination capability. During the last years we studied systematically the use of Fourier Transform Infrared (FTIR) spectroscopy as an alternative, powerful tool for quantifying VOCs in air. We calibrated the method for a set of compounds (styrene, acetone, ethanol and isopropanol) by using both laboratory and portable infrared spectrometers. The aim was to develop a new, and highly sensitive sensor system for VOCs monitoring. In this paper, we improved the setup performance, testing the feasibility of using a multipass cell with the aim of extending the sensitivity of our system down to the part per million (ppm) level. Considering that multipass cells are now also available for portable instruments, this study opens the road for the design of new high-resolution devices for environmental monitoring.

## 1. Introduction

The term volatile organic compounds refers to a group of toxic organic chemicals which have low boiling points and evaporate easily at room temperature (RT); therefore, they can rapidly spread in the environment, causing outdoor and indoor air pollution [1,2,3,4]. These compounds can be produced from natural processes, such as human and animal metabolic processes, plant and tree emissions, forest fires, biomass and carbon combustion, but they are mainly produced from anthropogenic processes, as by-products of various industrial and domestic activities including printing, building and storage of materials, food extraction and cooking [5,6]. In particular, styrene is the basis for polymeric resin and other solvents for cleaning, refining and polishing, largely produced in fiberglass manufacturing, and simultaneously present in the same workplace in combination with other by-products (acetone, ethanol, isopropyl alcohol, methanol, etc.). Nevertheless, they are considered responsible for several casualties [3]. Recent epidemiological studies have shown that in the worst cases exposure (accidental and/or chronic) to VOCs, which can occur through inhalation, ingestion and dermal contact, may cause respiratory illness, neurocognitive impairment and cancer [7,8,9]. Although the risk of exposure to several families of VOCs, as single compounds and/or in combination with each other, is recognized as harmful by the authorities, the legislation on professional monitoring and exposure limits is still lacking, and in some cases, such as for styrene, the legislation does not impose any legal obligations on manufacturers.

Therefore, the design of high-resolution, portable and reliable indoor and outdoor air quality monitoring tools is an important task to prevent overexposure to VOCs, in particular in indoor environments such as working sites.

Nowadays, different analytical tools can be used for detecting VOCs in the atmosphere, i.e., gas semiconductor sensors [10,11,12,13,14,15] and micromechanical resonant sensors [16] with a high sensitivity, but also analytical instruments based on Photo-Ionization Detectors (PIDs). Nevertheless, these devices do not allow easy discrimination between different chemical agents. In contrast, analytical methods such as gas chromatography, mass spectroscopy and vibrational spectroscopies are able to accomplish this goal allowing both the detection of VOCs at very low concentrations and their reliable discrimination [11,17,18,19,20,21]. Despite this, they are typical laboratory-based techniques not designed for in situ or real-time monitoring and require expensive equipment and skilled operators. Among these techniques, vibrational spectroscopy, such as Terahertz (THz) and Infrared (IR), have been shown to be more efficient, particularly in discriminating between different substances with a high accuracy. Notably, the potential of Mid-Infrared (MIR) spectroscopy is related to the numerous absorption molecular lines of interest allowing the different VOCs to be recognized. This ensures a high selectivity and sensitivity for ambient-level detection (typically in the ppm range) of common hazardous air pollutants, which are typically strong IR absorbers. The real-time monitoring of indoor exposure represents a challenging issue, in particular in occupational and environmental safety and health security. Notable examples such as open-path FTIR and THz measurements [22,23,24] have demonstrated the ability of vibrational spectroscopy to analyze ambient air in situ and in real time, making it a good candidate for implementing portable devices.

In this work, we used FTIR spectroscopy in the MIR to selectively identify organic chemical compounds in air, starting from our previous study [21] where the general methodology was set up. After the first experiments, by combining a dedicated PID sensor with MIR spectroscopy we calibrated the IR technique for the quantitative analysis of a series of VOCs [21]: styrene, acetone, ethanol and isopropanol. Here, we extended the sensitivity of this technique by using a multipass gas cell. More specifically, the main aim of this work was to demonstrate the feasibility of gas monitoring down and possibly below the ppm domain. The final goal was to develop a robust quantitative tool to be implemented on portable devices. The availability on the market of compact multipass cells tailored to also fit on a portable IR spectrometer, similar to that described in [21] has opened the possibility for new monitoring strategies. To this purpose, here we tested a laboratory-based gas cell to calibrate the spectroscopic analysis of four model compounds. Binary and ternary mixtures were also characterized to address the discriminating capability of the method when different VOCs are present in the atmosphere.

## 2. Materials and Methods

### 2.1. Materials

We considered four liquid VOCs: styrene, acetone, ethanol and isopropanol purchased by Sigma Aldrich and Carlo Erba. Styrene (*C_8_H_8_*–Purity ≥99.0% Carlo Erba Reagents,) belongs to the family of aromatic hydrocarbons, and can be found in varnishes, detergents, propellants and acetone (*C_3_H_6_O*–Purity 99.5% Sigma Aldrich) is a ketone, commonly used as solvent in several industrial applications, such as polymer production, cosmetics, lacquers, cellulose acetate and varnishes. Finally, ethanol (*C_2_H_6_O*–Purity ≥99.8% Sigma Aldrich) and isopropanol (*C_3_H_8_O*–Purity ≥99.9% Sigma Aldrich) are alcohols and are both used as disinfectants and detergents, as additive solvents for cleaning optical and electronic components and in the cosmetic field.

### 2.2. Method

**Figure 1:**
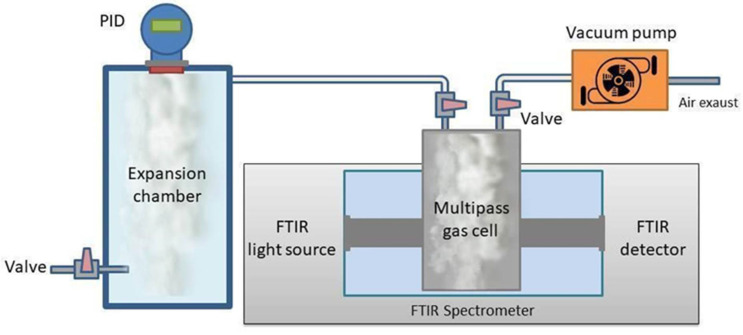
Schematic view of the setup used for the calibration experiments. The multipass gas cell, located in the sample compartment of a Vertex 70v Michelson interferometer, was connected to a sealed evaporation chamber. The PID system was installed on top of the evaporation chamber and was connected to a computer for real-time monitoring of evaporated VOCs.

FTIR measurements for the calibration of the multipass device were performed using the Bruker Vertex 70V interferometer at the Physics Department of Sapienza University in Rome. The schematic experimental setup is shown in Figure 1. It was similar to the portable device described in [21], with the exception of the gas cell, that in the present case was replaced by the GEMINI Mars Series Multi-Pass Gas Cells 2 L/10 M with a nominal path length of 10 m and a volume of 2 L. This multipass cell, which allowed us to achieve a high sensitivity in the absorbance measurements, was connected to a sealed evaporation chamber, where a commercial Photo-Ionization Detector (PID) sensor was installed for real-time monitoring of the evaporated VOC. The PID sensor (TA-2100 Styrene Detector from Mil-Ram Technology, Inc., Fremont, CA, USA) was calibrated for the detection of styrene in the range 1–100 ppm_v_, where ppm_v_ means parts per million volume. According to the manufacturer, this sensor has a sensitivity of 1 ppm_v_. Concerning the other VOCs, the reading of ppm_v_ was performed with a PID sensor normalized to a correction factor (CF from RAE, 2013 for a PID UV lamp at 10.6 eV) [25]. In this work, we used the following values for CF: 2.75 for acetone, 30 for ethanol, 15 for isopropanol and 1 for styrene. For these experiments, different amounts of liquid styrene, acetone, ethanol and isopropanol were introduced with a micropipette inside the evaporation chamber whose volume was ~0.6 l. The concentration was monitored against time using the PID sensor interfaced with a computer using LabVIEW^TM^ software. As soon as the countings provided by the PID were constant, indicating the attainment of the equilibrium condition within the chamber, corresponding to the maximum evaporation of the liquid, the left valve in Figure 1 was opened and the gas was transferred into the multipass gas cell inside the spectrometer. The flow was ensured by pre-evacuating the cell (vacuum pressure around a few mbar) closed by two 25 mm wide and 4 mm thick KBr windows, using a vacuum system (Edwards T-Station 85) constituted by a turbomolecular and a diaphragm vacuum pump. Spectra collection was started immediately after opening the connection between the expansion chamber and the multipass, together with the simultaneous recording of the PID readings. A total of 15 acquisitions were collected during the gas distribution in the whole volume, each spectrum being the average of 64 scans, with a nominal resolution of 2 cm^−1^ in the 400–5000 cm^−1^ frequency range. After few minutes, the spectra reached a constant intensity indicating an equilibrium condition between the expansion chamber and the cell. We then collected 10 spectra to be considered for the statistical data analysis. Basic operations such as baseline, spectral windows identification and peak integration were performed using OPUS™ 8.2 software (Bruker Optik, Germany). The linear fit to determine the calibration curves was performed with the ORIGIN PRO™ 9.0 software (OriginLab Corporation, USA).

## 3. Results and Discussion

### 3.1. Characterization and Statistical Analysis

**Figure 2:**
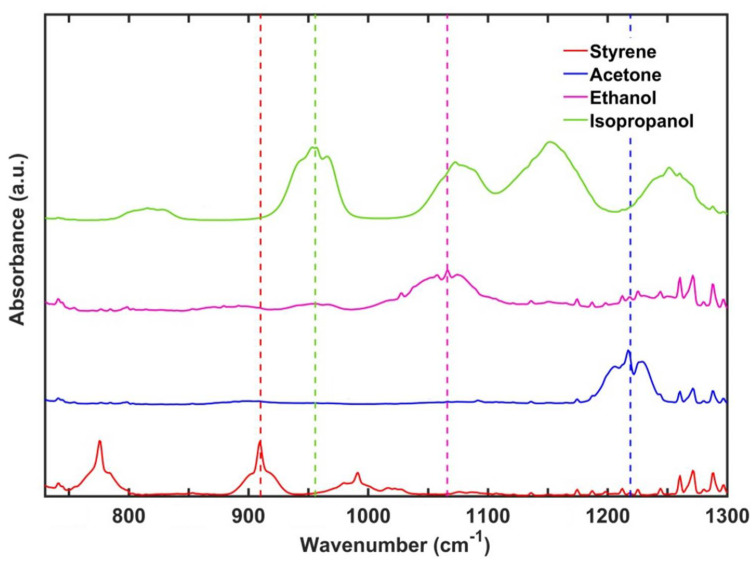
FTIR spectra of the studied VOCs in the MIR spectral region ranging between 750 and 1300 cm^−1^. Colored dashed lines highlight the absorbance peaks considered for the evaluation of the calibration curves of each VOC: styrene (red), acetone (blue), ethanol (pink) and isopropanol (green).

Figure 2 shows selected MIR absorption spectra of the investigated VOCs. The resulting spectra usually displayed several absorptions related to atmospheric H_2_O and CO_2_ in the 3000–4000 cm^−1^ and 1750–2100 cm^−1^ ranges and around 2400 cm^−1^, respectively. Beside these absorptions, in the windows around 1100 and 3100 cm^−1^ the spectra showed exclusively the typical absorption features characteristic of each VOC. In Figure 2, the region between 750 and 1300 cm^−1^ is highlighted together with the features selected as representative for each compound during the calibration. In the spectrum of styrene, the peak at 910 cm^−1^ was assigned to an out of plane bending of CH bonds in the aromatic ring [26,27] while in the same spectral range, acetone only showed a broad absorption centered at 1229 cm^−1^ due to CC_2_ antisymmetric stretching [28]. The ethanol band was centered between 1010 and 1100 cm^−1^ and it was associated with different overlapped vibrational modes (1027 cm^−1^ wagging of CH_3_, 1057 cm^−1^ antisymmetric stretching of CCO and 1089 cm^−1^ rocking of CH_3_ [29]. Finally, the isopropanol peak occurred at 953 cm^−1^ and it was assigned to the CH_3_ rocking mode [30]. These bands were chosen because they were relatively intense and well resolved and showed minimal overlaps with the bands of the other compounds.

In transmission IR spectroscopy, the absorbance of a specific band is related to the amount of the target molecule via the Beer–Lambert relationship [21]:

(1) A(ν)=l C ε(ν)

where A(ν) is the absorbance (adimensional), *l* is the optical path of the cell (in cm), *C* is the concentration of the molecule (in ppm_v_) and  ε(ν) (ppm_v_^−1^ cm^−1^) is the absorption coefficient. Absorbances (*A_i_*) were obtained by integrating the area of the characteristic bands for each VOC and averaging over ten spectra recorded sequentially. The uncertainty on the integrated absorbance was the standard deviation calculated on the repeated measurements performed at the same ppm_v_ concentration and estimated to be 0.01 cm^−1^. The VOCs concentrations in the multipass gas cell, expressed in ppm_v_, were calculated as reported in the Supporting Information. The uncertainty on the concentration measurements was established to be 1 ppm_v_ from the PID manufacturer and it was properly scaled following the error propagation equation.

### 3.2. Curve Calibrations for Styrene and Individual Interfering VOCs

**Figure 3:**
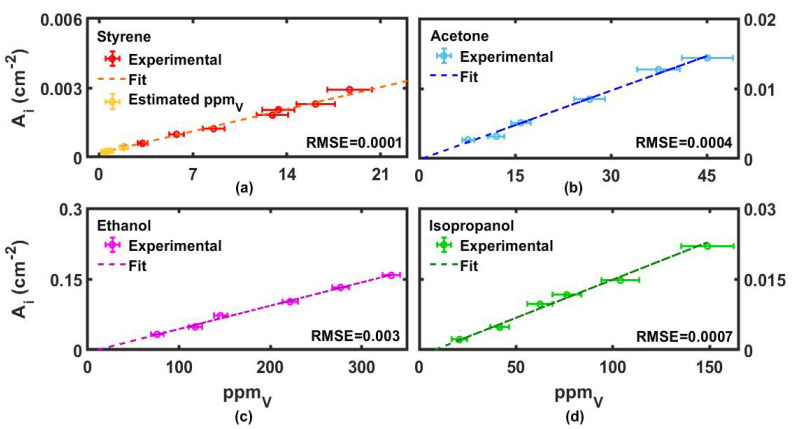
Calibration curves for styrene (**a**), acetone (**b**), ethanol (**c**) and isopropanol (**d**), referred to as the integrated absorbances A_i_ vs. ppm_v_. The experimental data are indicated by points and the fit curves by the dashed lines. In panel (**a**), we reported the A_i_ related to sub-ppm_v_ concentrations in yellow, indirectly estimated. The root-mean-square error (RMSE) was used for the estimation of the differences between the experimental values and the adopted linear model. (The integrated area errors are 0.1 × 10^−4^ cm^−2^).

We obtained the calibration curves for the four VOCs by correlating the integrated absorbance over pathlength for the characteristic VOC peak (*A_i_* was normalized with respect to the multipass optical path of 10 m) with the ppm_v_ estimated for each gas according to Equation (2). The relationship between absorbance and ppm_v_ was expressed using Equation (1) and the resulting curves are displayed in Figure 3, where the experimental data were linearly fitted by using the ORIGIN PRO™ software. The extrapolated fitting parameters, including slope and intercept of the calibration lines, are given in Table 1. The slope provided the absorption coefficient of the specific normal mode excited through IR radiation, whereas the intercept, likewise the integrated zero-concentration absorbance, was expected to approach zero.

To calculate the integrated absorbances, we selected the bands at 910 cm^−1^ for styrene, at 1229 cm^−1^ for acetone, at 1100 cm^−1^ for ethanol and at 953 cm^−1^ for isopropanol. The goodness of each fit was estimated through the root-mean-square error parameter (RMSE), reported in the corresponding panel of each calibration line. The inspection of Table 1 shows that the most intense absorption was obtained for ethanol; however, the absorption coefficients (slopes) associated to the different excited normal modes were all comparable. In addition, we have to underline here that the intercepts were all very close to zero, thus providing a good confirmation of the validity of the calibration.

As far as styrene is concerned, we tried to measure ppm_v_ values near the lower limit of PID detectability. Volumes of the order of μL of liquid styrene were put in the expansion chamber. Exploiting the capability of the multipass cell, we collected IR spectra and computed their corresponding integrated absorbances. Using the calibration line (shown in Figure 3a), we extrapolated the corresponding ppm_v_ values, which were: 0.46 ± 0.31, 0.76 ± 0.31 and 1.81 ± 0.32, well below the PID sensitivity and working linearity.

### 3.3. Comparison with Laboratory Detection Setups

**Figure 4:**
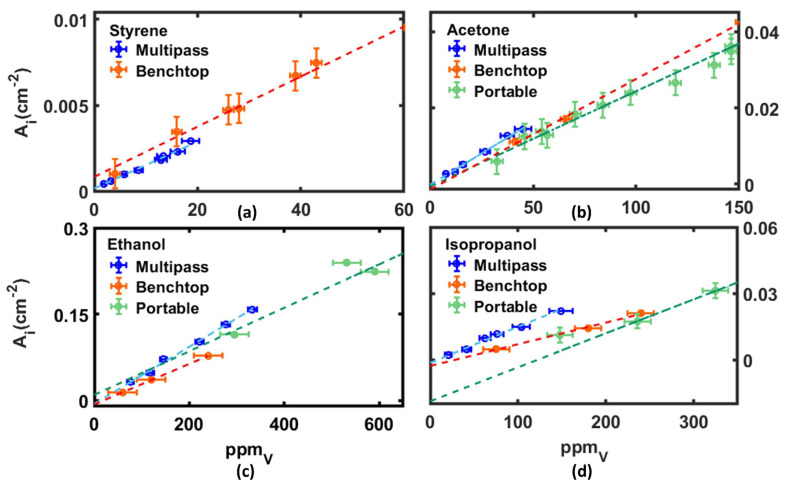
Enlargement view of the calibration of the integrated absorbance, normalized at the optical path, vs. ppm_v_ for styrene (**a**), acetone (**b**), ethanol (**c**) and isopropanol (**d**) obtained with the multipass (light blue line), benchtop (red line) and the portable device (green line), respectively. The whole calibration curves of the integrated absorbance vs. ppm_v_ obtained with the three setups are reported in Figure S3 of the Supporting Information. The integrated area errors were 0.1 × 10^−4^ cm^−2^ for multipass data (blue), 4∙10^−4^ cm^−2^ for benchtop data (red) and 0.001 cm^−2^ for portable data (green).

After the calibration of the system, as explained above, we compared the results obtained using the multipass cell with the results of the experiments performed with both a benchtop and a portable FTIR spectrometer (Figure S1) available from [11,21]. Figure 4 displays the calibration curves of styrene (Figure 4a), acetone (Figure 4b), ethanol (Figure 4c) and isopropanol (Figure 4d) obtained using all three set-ups: benchtop, portable FTIR and multipass. For the comparison we display the integrated absorbance, normalized with respect to the different optical path associated with each experimental setup, i.e., 27 cm for the benchtop system, 7 cm for the portable device and 1000 cm for the multipass layout.

For the sake of comparison, either the slope or the intercept of the calibration curves needed to be considered. For any VOC, the slope was expected to be the same, within the uncertainty for any experimental setup, because the absorption coefficient was independent of the specific experimental device or layout employed to perform the experiment. The plots of Figure 4 show that the intercept relative to the multipass was systematically very close to zero indicating that this device indeed provided the data with a better sensitivity. This behavior is easily explained by considering that, even at low VOCs concentration, with the multipass setup, thanks to the longer optical path length, the absorption bands (see Equation (1)) were more intense and the signal-to-noise ratio (S/N) was higher, ensuring a more accurate measurement. 

### 3.4. Quantitative Analysis of Binary and Ternary Mixtures of VOCs

One of the main advantages of using IR spectroscopy for VOCs detection is the possibility to discriminate, with a high degree of confidence, different chemical species, thanks to their different spectral features (see Figure 2), and to extrapolate their relative concentration in ppm_v_ from their integrated absorbance at the characteristic peaks using the calibration curves (Section 3.2). We tested the detectability of gas compositions on both binary and ternary mixtures. We prepared three binary solutions mixing volumes of acetone/ethanol (60:40 volume ratio), styrene/acetone (70:30), styrene/ethanol (70:30) and one ternary solution mixing styrene/acetone/ethanol (45:35:20). We injected the liquid solution into the expansion cell and waited for the equilibrium condition (liquid–vapor) by monitoring the gas concentration using the PID sensor.

**Figure 5:**
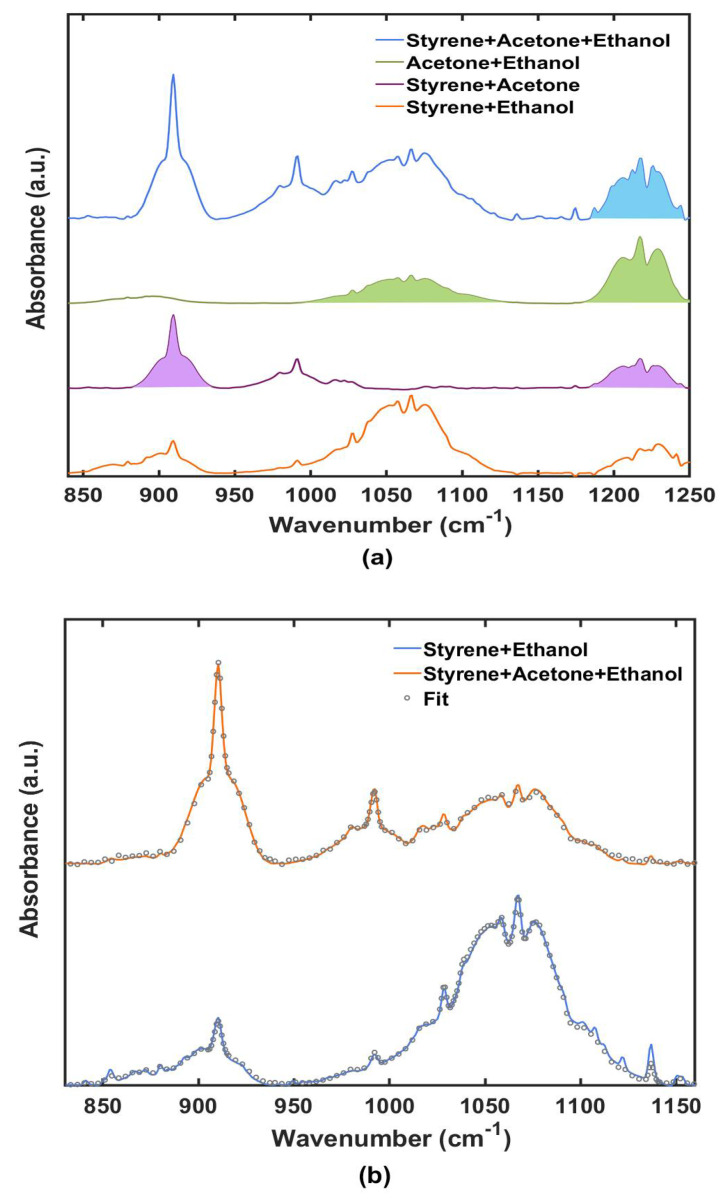
(**a**) Spectra of mixtures. Colored areas were calculated with the integration method of the OPUS™ 8.2 software. (**b**) Fit of styrene and ethanol absorbance peaks in the range 830–1160 cm^−1^ for a mixture of styrene+ethanol (orange) and for a ternary mixture (blue).

When the interaction among the components can be considered negligible, the net absorbance of a gaseous mixture can be considered as the sum of the contribution of the linear absorbance of each component [31,32]. This is known as the multiple absorbers approach:

(2) $$A_{mix}^{exp}=\underset{i}{\sum }A_{i}$$

where $A_{mix}^{exp}$ is the experimental mixture absorbance and $A_{i}$ is the estimated absorbance of pure components, due to each species *i* at the same pressure and temperature conditions. Observing Figure 5a, the mixtures of styrene/acetone and acetone/ethanol showed absorption bands clearly distinguishable and separated, so we simply integrated the area of their characteristic peaks, as discussed above. On the other hand, styrene and ethanol showed non negligible peak overlaps in the 840–920 cm^−1^ and 980–1010 cm^−1^ ranges, so it was not possible to simply integrate in the frequency window of interest for that VOC. It was necessary to fit the mixture spectrum in order to extrapolate the single VOC contribution to the absorbance. We looked at the mixture spectrum as the sum of individuals, each one multiplied by a factor representing the VOC concentration:

(3) Xmixture=α Xethanol+β Xstyrene

Using the Matlab function fminsearch we found the most proper *α* and *β* coefficients to satisfy this equation (see Supporting Information for mathematical specifications). We performed this calculation separately for the two frequency ranges, 830–945 cm^−1^, where the styrene peak was present, and 946–1161 cm^−1^, where there was the ethanol peak, therefore obtaining α from the second one and  β  from the first one.

Mixture spectra are shown in Figure 5a. Colored areas are the ones integrated with the single-peak method already discussed in the calibration sections, while the range 830–1160 cm^−1^ for the ternary and the styrene + ethanol mixtures was analyzed with the fitting procedure and the results are reported in Figure 5b. In Table 2, we report the concentrations in ppm_v_ for the individual VOCs of the four mixtures, obtained from the integrated areas and the calibration curves.

The sum was calculated adding the concentrations in ppm_v_ as extrapolated from the integrated areas, each one divided by the relative CF. PID lecture instead was the concentration in ppm_v_ measured directly by the instrument. An inspection of the final results (last two columns in Table 2) shows an excellent agreement between the concentrations obtained by summing up the contribution of the individual VOCs and the PID readings that represents the total (unresolved) gas concentration. The two values were coherent, showing the goodness of fit procedure and the capability of the multipass setup to determine mixture components. The data in Table 2 thus provide good evidence for the reliability of calibration used to extrapolate the single ppm_v_ from the IR spectrum collected from the mixture.

## 4. Conclusions

In the present work we provided a new, improved calibration curve for the IR spectroscopic detection for a series of gaseous compounds of environmental and occupational interest. The used technique was based on the combination of an IR Michelson interferometer and a multipass gas cell. In particular, we calibrated the quantitative analysis in the MIR spectral region for four different VOCs (styrene, acetone, ethanol and isopropanol) focusing on its advantages compared to portable and benchtop devices used previously [11,21], in terms of both detection sensitivity and accuracy. The longer optical path (10 m) inside the multipass cell ensured an excellent S/N ratio, thus allowing the discrimination of less intense vibrational bands, overcoming the limitations affecting the VOCs quantification when using conventional set ups. 

Styrene, acetone, ethanol and isopropanol were investigated individually in order to obtain accurate calibration curves. The four gas-phase VOCs exhibited linear relationships between the integrated absorbance of the specific vibrational bands and the corresponding concentrations provided by the independent measurement using a styrene-calibrated PID. The use of the calibration curves provided a reliable method to obtain sub-ppm_v_ concentrations well below the PID detection capability, as observed, in particular, for styrene.

In order to mimic real “conditions” present in indoor environments, binary and ternary VOCs mixtures were also prepared and analyzed. The obtained results underlined the capability of our setup to discriminate the different chemical species present simultaneously in the atmosphere, thanks to their unique spectral features. In particular, for measuring mixtures whose total concentration fell within the PID detection range, our spectroscopic approach allowed the concentration of each individual component otherwise not measurable with the PID sensor to be extracted. The results of our study thus pave the way toward the design of improved compact (and portable) air-quality monitoring systems capable of discriminating VOCs present simultaneously in the atmosphere and quantifying them down to the sub-ppm_v_ range.

## Figures and Tables

**Table 1:** Slope and intercept associated with the linear fit of styrene, acetone, ethanol and isopropanol.

VOCs	Slope (cm^−2^)	Intercept (cm^−2^)
Styrene	(1.3 ± 0.07) × 10^−4^	(0.17 ± 0.04) × 10^−3^
Acetone	(3.3 ± 0.2) × 10^−4^	(−0.2 ± 0.4) × 10^−3^
Ethanol	(5.0 ± 0.2) × 10^−4^	(−5.5 ± 3.7) × 10^−3^
Isopropanol	(1.6 ± 0.1) × 10^−4^	(−1.3 ± 0.7) × 10^−3^

**Table 2:** Concentration values (ppm_v_) extrapolated from the integrated areas of the absorbance peaks for different mixtures. The sum of the calculated values can be compared with the PID lecture.

	Styrene (ppm_v_)	Ethanol (ppm_v_)	Acetone (ppm_v_)	Sum * (ppm_v_)	PID Lecture (ppm_v_)
styrene + ethanol	(3.3 ± 0.3)	(27.2 ± 7.4)	//	(4.2 ± 0.6)	(5.3 ± 0.5)
styrene + acetone	(4.64 ± 0.38)	//	(10.3 ± 1.2)	(8.4 ± 0.8)	(11.1 ± 1.0)
acetone + ethanol	//	(20.2 ± 7.4)	(27.1 ± 2.1)	(10.5 ± 1.0)	(7.4 ± 0.7)
styrene + acetone + ethanol	(6.6 ± 0.5)	(14.3 ± 7.4)	(5.6 ± 1.3)	(9.1 ± 1.2)	(9.9 ± 0.9)

* Sum was calculated by summing up the concentrations scaled using the styrene calibration to make the comparison with the initial PID readings possible, i.e., Sum = ∑ (Concentration (ppm_v_)/ CF).

## Data Availability

The data presented in this study are available on request from the corresponding author.

## Supplementary Materials

The following supporting information can be downloaded at: https://www.mdpi.com/article/10.3390/s22155624/s1.
