# Supplementary material for: High Sensitivity Monitoring of VOCs in Air through FTIR Spectroscopy Using a Multipass Gas Cell Setup
Source: Sensors (Basel). 2022 Jul 27;22(15):5624. doi: 10.3390/s22155624 (PMC9370991; doi:10.3390/s22155624)
Supplement: Supplementary file 1 [file sensors-22-05624-s001.zip › sensors-1809398-supplementary.pdf]

sensors

High Sensitivity Monitoring of VOCs in Air through FTIR Spectroscopy Using a Multipass Gas Cell Setup

Supporting Information

S1: PORTABLE AND BENCHTOP SETUPS
S2: PPMV DEFINITION AND DISTRIBUTION
S3: COMPARISON WITH LABORATORY DETECTION SETUPS
S4: FITTING PROCEDURE IN MATLAB

S1: Portable and benchtop setups

Calibration benchtop Fourier-transform infrared (FTIR) measurements were performed using a Bruker Vertex 70 interferometer. The spectrometer was coupled with a commercial Photo-Ionization Detector (PID) (TA-2100 Styrene Detector from Mil-Ram Technology, Inc., Fremont, CA, USA) calibrated for the detection of styrene in the range 10–100 ppm_v. According to the manufacturer, this sensor has a sensitivity of 1 ppm_v. A schematic drawing of the experimental set-up is shown in Figure S1a. The PID system was mounted on top of the spectrometer chamber and was connected to an external computer for data collection and analysis; the sample compartment was separated by IR transparent windows from the rest of the optical system. Selected amounts of liquid VOCs were placed on a Corning® Petri dish located at the base of the spectrometer sample chamber (IR light path length 28 cm) and allowed to evaporate completely. The gas concentration in the chamber was monitored simultaneously with the PID on top, every 1 s, and by collecting FTIR spectra in continuous mode, every 30 s for the first hour and then every 60 s. Each experiment lasted from 1 to 3 h. FTIR spectra were obtained averaging 32 scans, with a nominal resolution of 4 cm^{-1} in the 400–5000 cm^{-1} spectral range.

Sensors **2022**, *22*, 5624. https://doi.org/10.3390/s22155624 www.mdpi.com/journal/sensors

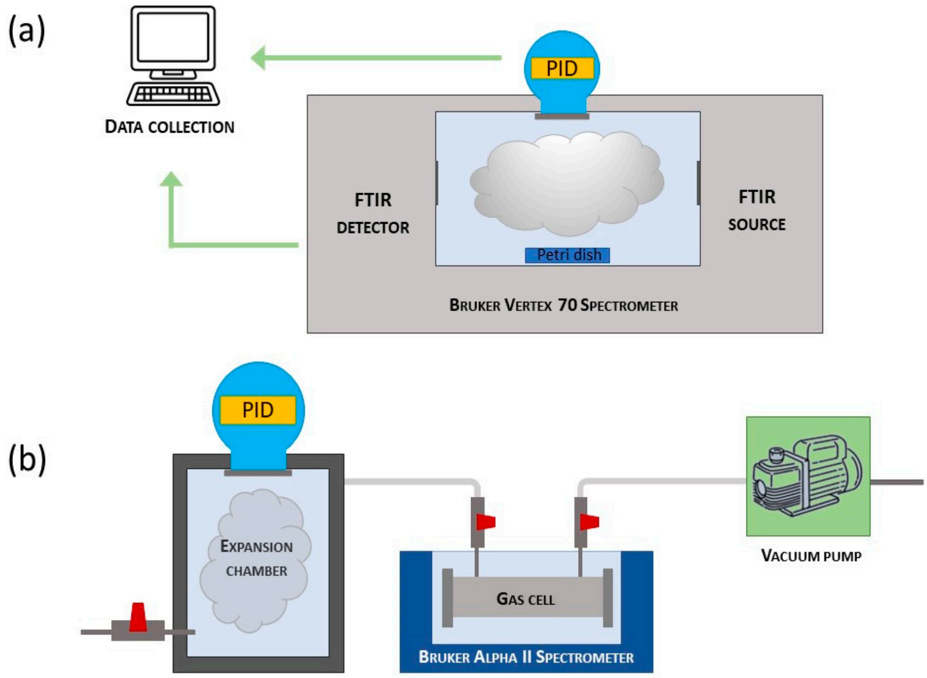

Figure S1. Schematic view of the benchtop (**a**) and portable (**b**) setups used for the calibration experiments and reported in our previous work [21].

The collection of each spectrum required around 30 s. Considering the main objective of this work, we selected several common interfering VOCs associated with styrene in workplaces to be tested alone or mixed with styrene. Detection of interfering VOCs was performed using the same PID optimized for styrene. Therefore, the measured values (i.e., equivalent ppm_v) were converted into the real concentrations of these gasses using appropriate correction factors (for a PID UV lamp at 10.6 eV). For the calibration of the portable device, we used a Bruker Alpha II spectrometer equipped with a gas cell with a path length of 7 cm (cell volume ~ 0.01 L). The gas cell was connected to a sealed evaporation chamber, where the PID sensor was also installed for real-time monitoring of evaporated VOCs. Figure S1b shows a schematic layout of this configuration. For these experiments, different amounts of liquid ethanol, acetone and isopropanol were introduced with a pipette inside the evaporation chamber (chamber volume ~ 0.6 L), and the concentration monitored against time using the PID sensor. As soon as the PID readings indicated the complete evaporation of the liquid within the chamber, the gas was transferred into the gas cell of the spectrometer. The flow was ensured by pre-evacuating the cell (vacuum pressure around 50 mbar) closed by two 5 mm thick KBr windows, using a vacuum pump (Figure S1b). Spectra were collected averaging 16 scans, with a nominal resolution of 4 cm^{-1} in the 4500–500 cm^{-1} range, at the scan velocity of 7.5 kHz. The reduced number of scans with respect to those used for the measurements with the Vertex allowed the time required to collect a spectrum (30 s) to be kept constant.

In order to compare the detection sensitivity of the three experimental setups employed in this work and in the previous one [21], Figure S2 displays three spectra of roughly the same quantity of ethanol gas (around 200–300 ppm_v) collected with a conventional benchtop device (in orange), a portable device equipped with a short-path gas cell (in green) and a benchtop device equipped with a multipass cell (in blue). On one hand, the most intense peak around 1050 cm^{-1} was very distinguishable and had the same shape for the three setups. On the other hand, the less intense peak around 875

cm^{-1} was well resolved only in the gas cell, revealing that, as expected, the multipass spectrum was a couple of orders of magnitude more intense than the others.

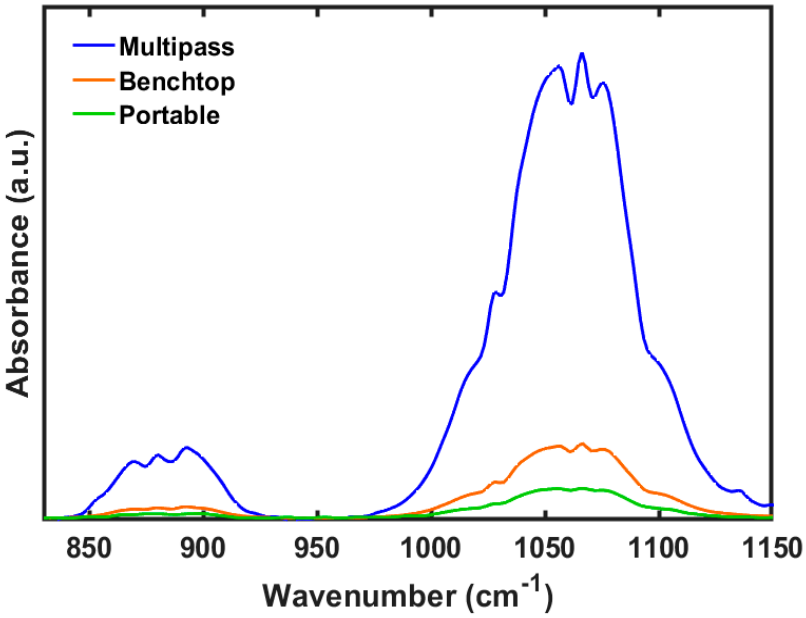

Figure S2. Ethanol FTIR spectra in the 800–1150 cm^{-1} spectral range collected using a conventional benchtop device (28 cm path, in blue), a benchtop device equipped with the multipass cell (10 m path, in orange), and a portable device equipped with a short-path gas cell (7 cm FTIR path, in green).

S2: ppm_V definition and distribution

The PID reading was expressed in units of ppm_v, i.e. parts per million by volume, related to molar concentration (in mol/m^3) as follows:

$$\text{Conc}\ (\text{ppm}_v) = 24.46 \cdot \text{Conc}\ (\text{mg/m}^3)\ /\ \text{molecular weight}\ (\text{g/mol}) \tag{S1}$$

where 24.46 is air molar volume at 25°.

The PID readings in ppm_v at the beginning of the experiment were referred to the gas concentration in the volume of the evaporation chamber (V_1). When the evaporation chamber was connected to the multipass gas cell, the gas expanded in the new volume (V_1+V_2), as shown in Figure 1. Thus, the concentration of the gas molecules was evaluated as follows:

$$\text{C} = \text{ppm}_{V1} \cdot \text{CF} \cdot \text{V1}\ /\ (\text{V1} + \text{V2}) \tag{S2}$$

where ppm_{v1} is the PID reading at the beginning of the experiment, V_1 is the volume of the evaporation chamber, V_2 is the volume of the multipass gas cell and CF is the factor used to convert the concentration provided by the PID and referred to styrene (the PID was factory-calibrated for styrene) to the concentration of other VOCs. CFs were experimentally measured with the procedure described in the PID handbook [25]. Here CFs were provided for different conditions of temperature, humidity, and detector voltage. In this work we used the following CFs: 2.75 for acetone, 30 for ethanol, 15 for isopropanol and 1 for styrene (CF from RAE, 2013 for a PID UV lamp at 10.6 eV). The uncertainty on the concentration measurements was established to be 1 ppm_v from the PID manufacturer and it was properly scaled following the error propagation equation.

S3: Comparison with laboratory detection setups

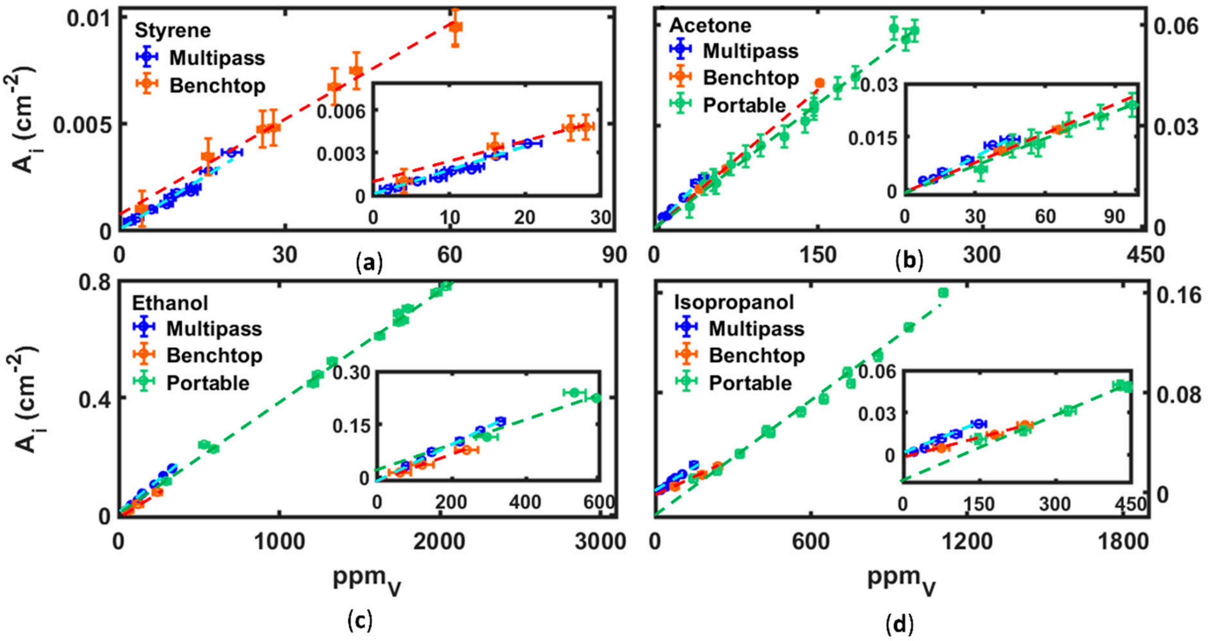

Figure S3. Calibration of the integrated absorbance, normalized at the optical path vs. ppm_v for styrene (**a**), acetone (**b**), ethanol (**c**) and isopropanol (**d**) obtained with the multipass (light blue line), benchtop (red line) and the portable device (green line), respectively. The plots were reported in a larger range compared to the insets, which depicted a zoom of each calibration curve, already shown in Figure 4.

S4: Fitting procedure in Matlab

In the VOCs mixtures, in order to discriminate contributions to the whole IR absorbance spectrum of individual elements, an iterative method was employed using the Matlab function *fminsearch*. Looking at the mix spectrum as the linear combination of individual ones, the Matlab script found the values of parameters $\boldsymbol{\alpha}$ and $\boldsymbol{\beta}$ which minimized the value of function f defined as follows:

$$f = \sum_i | (X_{mix} - \alpha X_{ethanol} - \beta X_{styrene}) | \quad \text{(S3)}$$

where X_{mix}, $X_{ethanol}$ and $X_{styrene}$ are the arrays of experimental spectra and the sum is intended on the array index. The procedure was performed separately on two spectral ranges of interest, ranges 830–945 cm^{-1} and 945–1161 cm^{-1} and therefore styrene and ethanol minimized spectra were obtained.
